# *MdATG5a* ‐mediated autophagy improves apple performance under low light

**DOI:** 10.1111/tpj.71108

**Published:** 2026-09-10

**Authors:** Weijia Xiang, Yue Fang, Yanghao Long, Qiuxian Zhou, Ziyan Li, Xiaoqing Gong, Fengwang Ma

**Affiliations:** ^1^ State Key Laboratory for Crop Stress Resistance and High‐Efficiency Production/Shaanxi Key Laboratory of Apple, College of Horticulture Northwest A&F University Yangling 712100 Shaanxi China

**Keywords:** apple, *MdATG5a*, low light, carbon and nitrogen metabolism, photosynthesis

## Abstract

Apples are sun‐loving plants, and excessive canopy closure in orchards leads to poor ventilation and reduced light penetration, ultimately limiting yield and fruit quality. Autophagy is a conserved mechanism for recycling cellular materials and energy in eukaryotes and is known to participate in multiple stress responses. However, whether autophagy contributes to low‐light adaptation remains largely unclear. Here, we show that *MdATG5a* enhances the tolerance of apple plants to low‐light conditions. Under low light, *MdATG5a*‐overexpressing (OE) plants exhibited higher photosynthetic capacity, greater biomass, and increased carbohydrate accumulation compared with wild‐type (WT) plants. During acclimation to low light, OE plants enhanced light harvesting and promoted the turnover of photosynthetic proteins by increasing leaf area and the leaf nitrogen content. Relative to WT, OE plants displayed a lower light compensation point and light saturation point, reduced dark respiration rate, higher net photosynthetic rate and stomatal conductance, and less structural and functional damage to chloroplasts and photosystem II. Metabolite profiling further revealed that OE plants had more stable levels of flavonoids and carbohydrates and their derivatives. OE plants also showed higher autophagic activity than WT plants, and pharmacological inhibition of autophagy abolished the growth advantage of OE plants under low light. Moreover, we found that *MdATG5a* is positively regulated by MdPIF7, a key transcription factor in shade‐related light signaling. Together, these findings indicate that *MdATG5a*‐mediated autophagy, regulated by MdPIF7, is crucial for the adaptation of apple to low light by enhancing autophagic flux, improving photosynthetic performance, and stabilizing metabolic homeostasis.

## Significance statement

Low light severely reduces apple yield and quality, while its adaptive molecular mechanisms are unclear. We found MdPIF7‐activated, MdATG5a‐mediated autophagy improves apple low‐light tolerance through three key routes: boosting photosynthesis, reshaping carbon‐nitrogen allocation and maintaining cellular metabolic balance. Our results confirm autophagy acts as a core adaptive strategy for woody fruit plants to survive long‐term weak light stress.

## INTRODUCTION

Light is a key environmental factor regulating plant growth and development (Gao et al., 2021). Light quality, light intensity, and photoperiod influence plant defense responses and signaling, and light intensity and light quality are the primary constraints of photosynthesis and carbohydrate synthesis (Tian et al., 2017). Under low light, plant biomass is markedly reduced, and plants typically exhibit shade‐acclimation traits such as increased leaf area, more horizontal leaf angles, and reduced structural robustness (Hirano et al., 2019; Wu et al., 2018; Xu et al., 2007). Plants also adjust photosynthetic parameters to adapt to low light, including the light saturation point (LSP), light compensation point (LCP), dark respiration rate (Rd), and apparent quantum efficiency (AQE). These adjustments enable plants to satisfy growth demands at lower irradiance and improve light‐energy use efficiency by maintaining carbon (C) balance (Shu et al., 2016; Zhang, Ge, et al., 2022).

Apple trees are sun‐loving and require approximately 2200–2800 sunshine hours per year, making adequate canopy ventilation and light penetration crucial for their growth and fruit quality. In the Loess Plateau in Northwest China, the largest apple production area in China, modern dwarf and high‐density planting systems have been promoted in recent years to enhance the local apple industry (Ma, 2023; Chen et al., 2025). However, environmental constraints have limited the full implementation of these systems, with arborized cultivation still widely used, resulting in excessive canopy closure, poor ventilation, and reduced light transmission. Prolonged rainy periods in autumn further reduce sunshine during fruit maturation. Improving the efficiency of light‐energy utilization in apple plants is thus essential for optimizing apple production in this region.

Low light decreases the turnover rates of photosynthetic pigments and enzymes and reduces the efficiency of light‐energy conversion (Franklin, 2008). One prominent effect of low light is the decline in ribulose‐1,5‐bisphosphate carboxylase/oxygenase (Rubisco) activity, which leads to insufficient CO_2_ assimilation (Shafiq et al., 2021). Under high‐density planting, the photosynthetic capacity of maize is reduced, and chloroplast ultrastructure is impaired (Ren et al., 2017). Zheng et al. (2022) reported that the reduction in the photosynthetic rate of spike leaves under low light is driven mainly by weakened photosynthetic activity of chloroplasts rather than stomatal limitations. Photosystem II (PSII) is also highly susceptible to damage under low‐light stress, resulting in reduced electron transport and ATP synthesis. In cigar tobacco, low light reduces the maximum photochemical quantum yield (Fv/Fm) and the efficiency of non‐cyclic electron flow of PSII, indicating damage to the photosynthetic structure (Khalekuzzaman et al., 2015; Wu et al., 2021). Shaded maize leaves exhibit similar changes and increased non‐photochemical quenching coefficient (NPQ), suggesting that reduced photosynthetically active radiation inhibits photochemical reactions and that non‐radiative energy dissipation is insufficient for preventing photodamage (Sui et al., 2012).

When light is sufficient, leaf photosynthetic capacity is enhanced, supporting the development of a more extensive root system to acquire nutrients and water for growth (Siddiqui et al., 2018). Numerous studies have shown that low light decreases the expression of photosynthetic products and genes associated with the Calvin cycle as well as sugar and starch synthesis (Hu et al., 2017; Zhou et al., 2022). In oil tea, limited photosynthetic C assimilation under low light restricts C export and carbohydrate synthesis, thereby hindering starch and sugar alcohol accumulation (Wu et al., 2022). At the same time, low‐light‐induced reductions in carbohydrates decrease belowground nutrient demand by the roots, leading to a greater allocation of dry matter to aboveground organs to support photosynthesis (Wu et al., 2022). In maize, increased planting density reduces the light intercepted by lower leaves and inhibits photosynthesis, which, in turn, alters dry matter partitioning; more biomass is allocated to stalks and leaves than to kernels under low light (Zheng et al., 2022). Shade‐tolerance studies have also shown that low light significantly suppresses rice photosynthesis, resulting in inadequate C reserves; thus, reducing respiratory consumption is essential for sustaining rice growth under low light (Valladares & Niinemets, 2008).

Autophagy is a key intracellular degradation and recycling pathway in eukaryotic cells and is essential for plant development and stress responses (Han et al., 2015). Under adverse conditions, autophagy removes damaged cellular components and redistributes the resulting products to enhance stress tolerance (Che et al., 2022). Excess light energy increases the accumulation of reactive oxygen species (ROS); autophagy mutants are more sensitive to high light intensity and exhibit more severe leaf damage and ROS accumulation (Goto‐Yamada et al., 2023). Drought stress increases autophagy activity in apple, wheat, and rice (Rana et al., 2012; Xiang et al., 2024; Zhang et al., 2020). C, N, and phosphate starvation all induce autophagy in plant cells (Ellström et al., 2015; Lin et al., 2023; Pei et al., 2014). Insufficient light limits energy synthesis in plants and promotes premature leaf senescence, making photosynthetic activity and carbohydrate reserves critical for plant growth and survival (Baena‐González et al., 2007; Moraes et al., 2019). In Arabidopsis, *atg* mutants display stunted growth and reduced amino acid levels under C starvation (Avin‐Wittenberg et al., 2015). Seedlings of autophagy mutants *atg2*, *atg5*, *atg7*, and *atg9* germinated in the dark show delayed chloroplast development after light exposure (Wijerathna‐Yapa et al., 2021). In lotus, Li et al. (2024) found that shade elevates *NnSnRK1* levels, activates *NnATG1*, and induces the formation of numerous autophagic vesicles; however, excessive autophagy triggers autophagic cell death and bud abortion. Shade has also been shown to induce a starvation‐like response and promote autophagy, while concurrently activating phytochrome‐interacting factors (PIFs) to upregulate genes involved in sterol biosynthesis (Ince et al., 2022). These two pathways independently drive growth adjustments that improve light capture and synergistically promote hypocotyl elongation under shade via autophagy (Ince et al., 2022).

Previously, we showed that the autophagy‐related gene *MdATG5a* improves nitrogen (N) use efficiency and drought tolerance in apple plants (Jia et al., 2021; Jia et al., 2022). *MdATG5a* enhances the photosynthetic capacity of apple plants under both drought and N deficiency, suggesting that it may be involved in regulating light‐energy use efficiency. Moreover, nutrient deficiency is considered one of the strongest inducers of autophagy (Feng et al., 2024; Kacprzak & Van Aken, 2022), and insufficient light limits carbohydrate synthesis, resulting in nutrient deprivation and promoting autophagy. However, whether autophagy really functions in the low‐light response is unclear. In this study, we systematically revealed the biological role of autophagy in apple plants responding to low‐light conditions, using transgenic apple plants overexpressing *MdATG5a* (*MdATG5a*‐OE). We found that, compared with wild‐type (WT) plants, *MdATG5a*‐OE apple plants exhibited superior growth and physiological adaptation to low light. This study suggests that manipulating *MdATG5a*‐mediated autophagy could improve light‐use efficiency in apple and aid the development of low‐light‐tolerant germplasm for orchard systems with suboptimal light conditions.

## RESULTS

### Effect of *MdATG5a* on the growth and leaf traits of apple plants under low light

**Figure 1:**
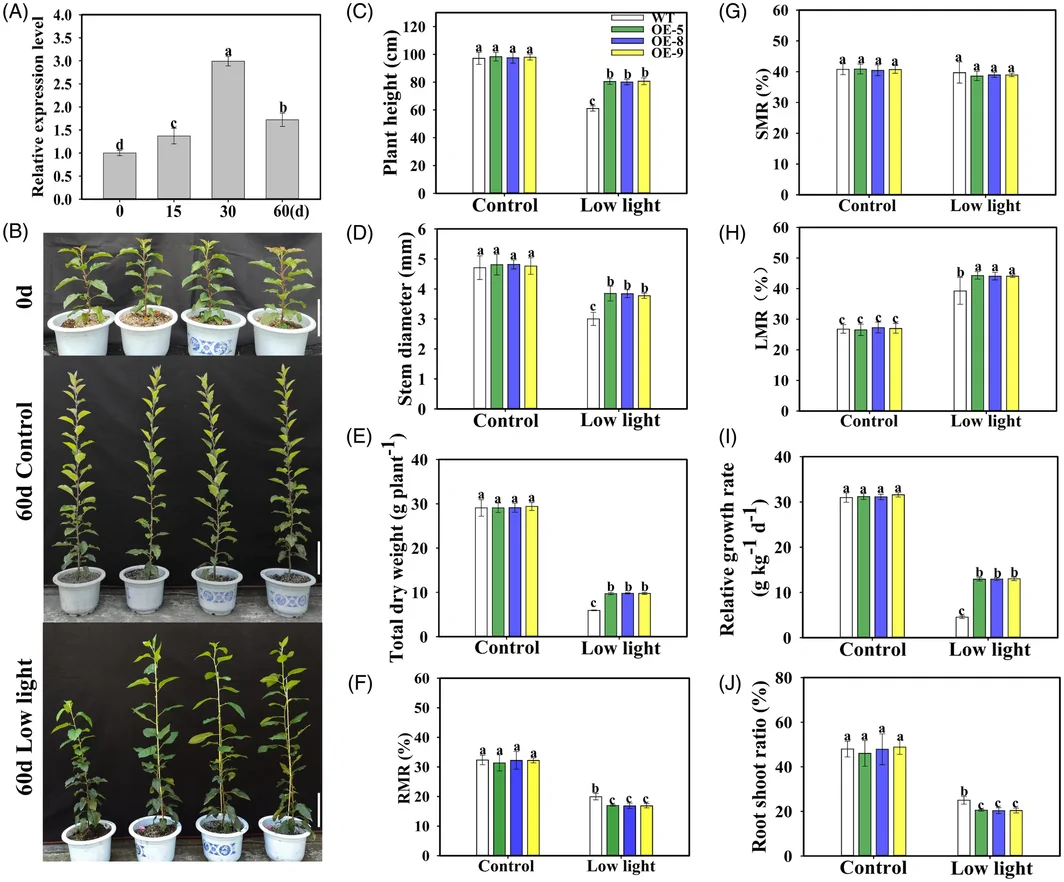
Effects of phenotype on *MdATG5a*‐OE and WT apple plants under low light. (A) The expression level of *MdATG5a* under low light. qPCR was carried out using the ‘GL‐3’ apple (*Malus domestica*) leaves after low‐light treatment. (B) Phenotypes of WT and *MdATG5a*‐OE transgenic apple plants after low‐light treatment. The scale bar = 15 cm. (C–J) Values of the plant height (C), stem diameter (D), total dry weight (E), RMR (%): the percentage of root dry weight to total plant dry weight (F), SMR (%): the percentage of stem dry weight to total plant dry weight (G), LMR (%): the percentage of leaf dry weight to total plant dry weight (H), relative growth rate (RGR) (I), root–shoot ratio (J) in WT and *MdATG5a*‐OE transgenic apple plants under low light. WT, wild type, here, we used ‘GL‐3’ apple (*M. domestic*), which was also used as explants in generating transgenic apple plants; OE‐5/8/9: transgenic apple plants overexpressed *MdATG5a*, which was obtained previously. Data are means ± SD. Different letters indicated that there were significant differences in Tukey's multiple test data (*P* < 0.05).

qPCR analyses revealed that *MdATG5a* was induced by low light in GL‐3 plants, suggesting that *MdATG5a* is potentially involved in low‐light responses (Figure 1A). To determine the function of *MdATG5a* under low‐light conditions, we conducted a 60‐day experiment using three *MdATG5a*‐OE lines (Jia et al., 2022). The phenotypes of the WT and OE plants did not significantly differ at the start of the experiment or after 60 days of natural light (Figure 1B, 0 and 60 days control). After low‐light treatment, all genotypes showed inhibited growth, with significantly decreased plant height and stem diameter. However, *MdATG5a*‐OE plants were less inhibited and accumulated higher biomass than WT plants (Figure 1B, 60 days low light; Figure 1C–E). RGR values were higher and root–shoot ratios were lower in OE plants than in WT plants after low‐light treatment (Figure 1I,J). We measured the proportion of each organ to the total dry weight in apple plants under low‐light treatment. The RMR of OE plants decreased more markedly than in WT plants after low‐light treatment compared with control conditions (Figure 1F). Conversely, the LMR increased more significantly in OE plants than in WT plants, while the SMR remained relatively unchanged (Figure 1G,H). Thus, under low light, apple plants tended to allocate more nutrients to the aboveground parts, especially to the leaves.

**Figure 2:**
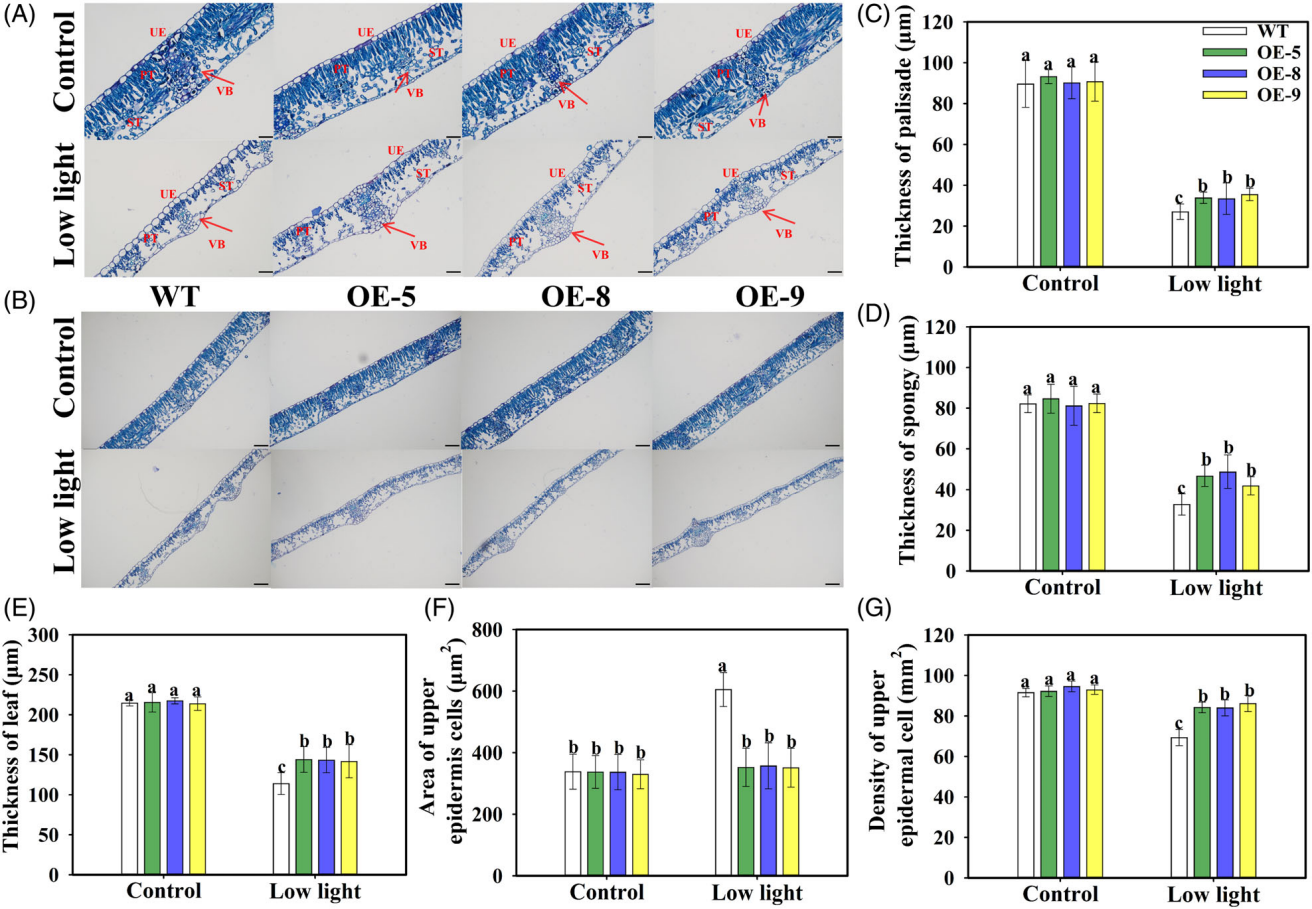
Effects of leaf tissue structure on *MdATG5a*‐OE and WT apple plants under low light. (A, B) Observation of cross‐sectional anatomy of apple leaves by microscope at 20 (A) and 10 (B) times. The scale bar in (A) =50 μm, the scale bar in (B) = 100 μm. (C–G) Values of the thickness of palisade (C), thickness of spongy (D), thickness of leaf (E), area of upper epidermis cells (F) and density of epidermal cell (G) in WT and *MdATG5a*‐OE transgenic apple plants under low light. UE refers to the upper epidermal; PT represents the palisade tissue; ST indicates the spongy tissue; VB stands for the vascular bundle. WT, wild type, here, we used ‘GL‐3’ apple (*M. domestic*), which was also used as explants in generating transgenic apple plants; OE‐5/8/9: transgenic apple plants overexpressed *MdATG5a*, which was obtained previously. Data are means ± SD. Different letters indicated that there were significant differences in Tukey's multiple test data (*P* < 0.05).

We then evaluated changes in leaves after low‐light treatment. In all genotypes, we observed decreases in leaf thickness, leaf number, and leaf dry weight, and increases in leaf length, leaf width, and leaf area. All these parameters were markedly higher in OE plants than in WT plants under low light (Table 1; Figure 2A,B,E). SLW, which reflects the leaf dry matter content per unit leaf area, was smaller in OE plants than in WT plants after low‐light treatment (Table 1). No differences in leaf tissue structure were observed between WT and OE plants under control conditions (Figure 2A,B, control). Low‐light stress caused the palisade and spongy tissues to become sparse and markedly decreased in thickness. These tissues were also thinner in WT plants than in OE plants (Figure 2C–E). Changes in the density of upper epidermal cells were consistent with these observations (Figure 2G). Additionally, the area of the upper epidermal cells in OE plants was nearly the same in the control and low‐light groups, but it was significantly larger in WT plants after treatment (Figure 2F). These results suggest that *MdATG5a* may aid light capture in apple plants by altering leaf tissue structure and thus mediating adaptation to low‐light environments.

**Table 1:** Leaf traits were measured from WT and *MdATG5a*‐OE plants after low‐light treatment

	Blade length (cm)	Blade width (cm)	Blade number (pcs)	Blade area (cm^2^)	Single blade dry weight (g plant^−1^)	Specific leaf weight (SLW)
Control						
WT	5.333 ± 0.14c	4.744 ± 0.136c	40 ± 1.58a	22.38 ± 0.649c	0.149 ± 0.007a	66 ± 1.4a
OE‐5	5.318 ± 0.258c	4.76 ± 0.08c	40.2 ± 1.92a	22.055 ± 0.704c	0.148 ± 0.009a	67 ± 3.06a
OE‐8	5.327 ± 0.121c	4.75 ± 0.13c	40 ± 2.24a	21.937 ± 0.867c	0.146 ± 0.01a	67 ± 2.63a
OE‐9	5.322 ± 0.1c	4.75 ± 0.08c	40 ± 2.45a	22.383 ± 1.967c	0.15 ± 0.022a	67 ± 3.77a
Low light						
WT	6.058 ± 0.155b	5.23 ± 0.12b	27.6 ± 1.14c	27.35 ± 2.156b	0.102 ± 0.01b	37 ± 1.86b
OE‐5	8.05 ± 0.075a	6.13 ± 0.13a	35.75 ± 0.96b	42.143 ± 0.163a	0.138 ± 0.007a	33 ± 1.62c
OE‐8	8.037 ± 0.126a	6.12 ± 0.13a	35.75 ± 1.26b	46.023 ± 4.983a	0.152 ± 0.024a	33 ± 1.74c
OE‐9	8.033 ± 0.306a	6.13 ± 0.08a	35.33 ± 0.5b	43.767 ± 1.564a	0.145 ± 0.011a	33 ± 1.27c

*Notes*: Control, plants without any treatment; Low light, plants with low‐light treatment. WT, wild type, here, we used ‘GL‐3’ apple (*M. domestic*), which was also used as explants in generating transgenic apple plants; OE‐5/8/9: transgenic apple plants overexpressed *MdATG5a*, which was obtained previously. Data are means ± SD. Different letters indicated that there were significant differences in Tukey's multiple test data (*P* < 0.05).

### Effect of *MdATG5a* on the photosynthetic characteristics of apple plants under low light

**Figure 3:**
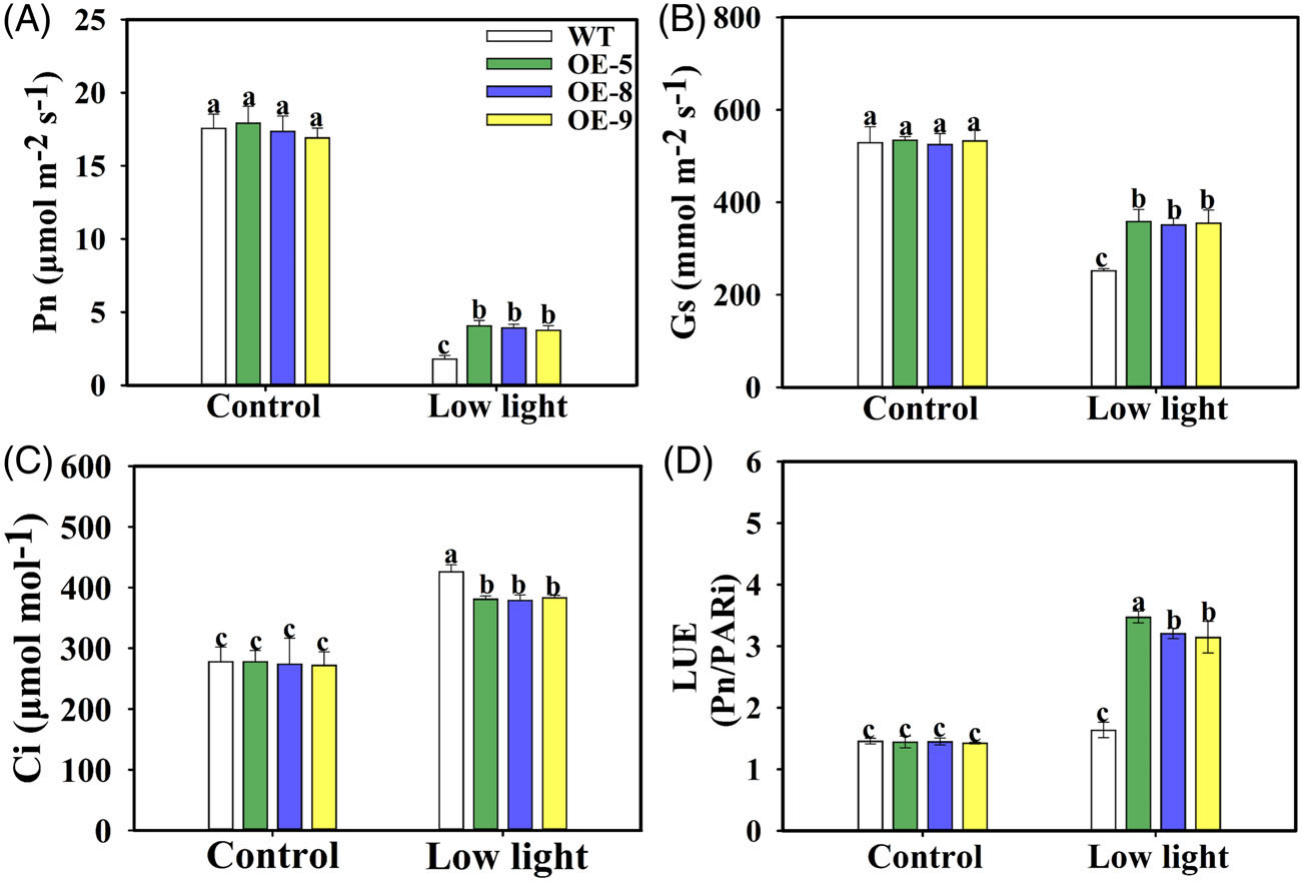
Effects of gas exchange parameters on *MdATG5a*‐OE and WT apple plants under low light. (A, B) The net photosynthesis rate (Pn) (A) and the stomatal conductance (Gs) (B) in WT and *MdATG5a*‐OE transgenic apple plants under low light. (C, D) The intercellular CO_2_ concentration (Ci) (C) and the unit light‐use efficiency (LUE) (D) in WT and *MdATG5a*‐OE apple plants under low light. WT, wild type, here, we used ‘GL‐3’ apple (*M. domestic*), which was also used as explants in generating transgenic apple plants; OE‐5/8/9: transgenic apple plants overexpressed *MdATG5a*, which was obtained previously. Data are means ± SD. Different letters indicated that there were significant differences in Tukey's multiple test data (*P* < 0.05).

We used light‐response curves and their fitting parameters to assess the photosynthetic efficiency of apple plants in response to low light. As shown in Table 2, under control conditions, Amax did not differ substantially between OE and WT plants. Low‐light treatment reduced Amax for all genotypes. However, the Amax of OE plants decreased by approximately 47.9% (43.7%, 50.3%, and 49.7% for OE‐5, OE‐8, and OE‐9, respectively), which was significantly lower than the reduction observed in WT plants (59.7%). Similarly, LSP, LCP, and Rd significantly decreased in all genotypes under low light, but these reductions were greater in OE plants than in WT plants (Table 2). AQE reflects the efficiency of light utilization by leaves. AQE in WT plants did not vary with or without low‐light stress, but it increased in OE plants under low‐light stress (Table 2). We found no significant differences in photosynthetic characteristics among all genotypes under control conditions (Figure 3). Low‐light stress inhibited the photosynthetic capacity of apple plants; however, Pn values were still approximately 2.24, 2.16, and 2.07 times higher in OE‐5, OE‐8, and OE‐9 than in WT plants, respectively (Figure 3A). A similar trend was observed for Gs (Figure 3B). Values of Ci increased under low light, and changes in Ci were more pronounced in WT plants (Figure 3C). LUE was higher in OE plants than in WT plants under low light (Figure 3D). These data indicate that *MdATG5a* overexpression in apple plants enhanced the photosynthetic capacity and resulted in higher light intensity utilization efficiency under low light.

**Table 2:** Effects of WT and *MdATG5a*‐OE plants on fitting parameters of light‐response curve under low‐light treatment

	AQE	Amax (μmol m^−2^ s^−1^)	LSP (μmol m^−2^ s^−1^)	LCP (μmol m^−2^ s^−1^)	Rd (μmol m^−2^ s^−1^)
Control					
WT	0.0358 ± 0.0023b	27.8172 ± 1.2553a	842.6384 ± 54.3094a	65.5597 ± 3.8191a	2.2798 ± 0.156b
OE‐5	0.0363 ± 0.0024b	26.7728 ± 0.4831a	833.3308 ± 31.3187a	65.753 ± 3.0736a	2.1353 ± 0.0986b
OE‐8	0.038 ± 0.0005b	27.654 ± 1.2803a	768.4488 ± 10.2175b	61.9171 ± 2.1534a	2.1969 ± 0.0339b
OE‐9	0.0378 ± 0.0016b	28.2202 ± 1.6507a	790.0227 ± 1.2391ab	62.9452 ± 0.6932a	2.4323 ± 0.0911a
Low light					
WT	0.037 ± 0.0011b	11.7211 ± 0.5268c	327.4051 ± 8.1566c	18.9967 ± 1.7719b	0.7209 ± 0.0772c
OE‐5	0.0483 ± 0.0096a	15.0653 ± 1.8437b	298.2536 ± 12.9221c	13.4851 ± 2.2323c	0.5243 ± 0.025d
OE‐8	0.0461 ± 0.001a	13.7443 ± 0.6172b	310.2227 ± 9.0160c	12.706 ± 0.3556c	0.57 ± 0.0141d
OE‐9	0.0462 ± 0.0021a	14.199 ± 8.2051b	311.6798 ± 7.2174c	13.227 ± 1.0971c	0.4917 ± 0.0238d

*Notes*: Control, plants without any treatment; Low light, plants with low‐light treatment. WT, wild type, here, we used ‘GL‐3’ apple (*M. domestic*), which was also used as explants in generating transgenic apple plants; OE‐5/8/9: transgenic apple plants overexpressed *MdATG5a*, which was obtained previously. Data are means ± SD. Different letters indicated that there were significant differences in Tukey's multiple test data (*P* < 0.05).

### *MdATG5a* promotes the energy conversion efficiency of apple plants under low light

**Figure 4:**
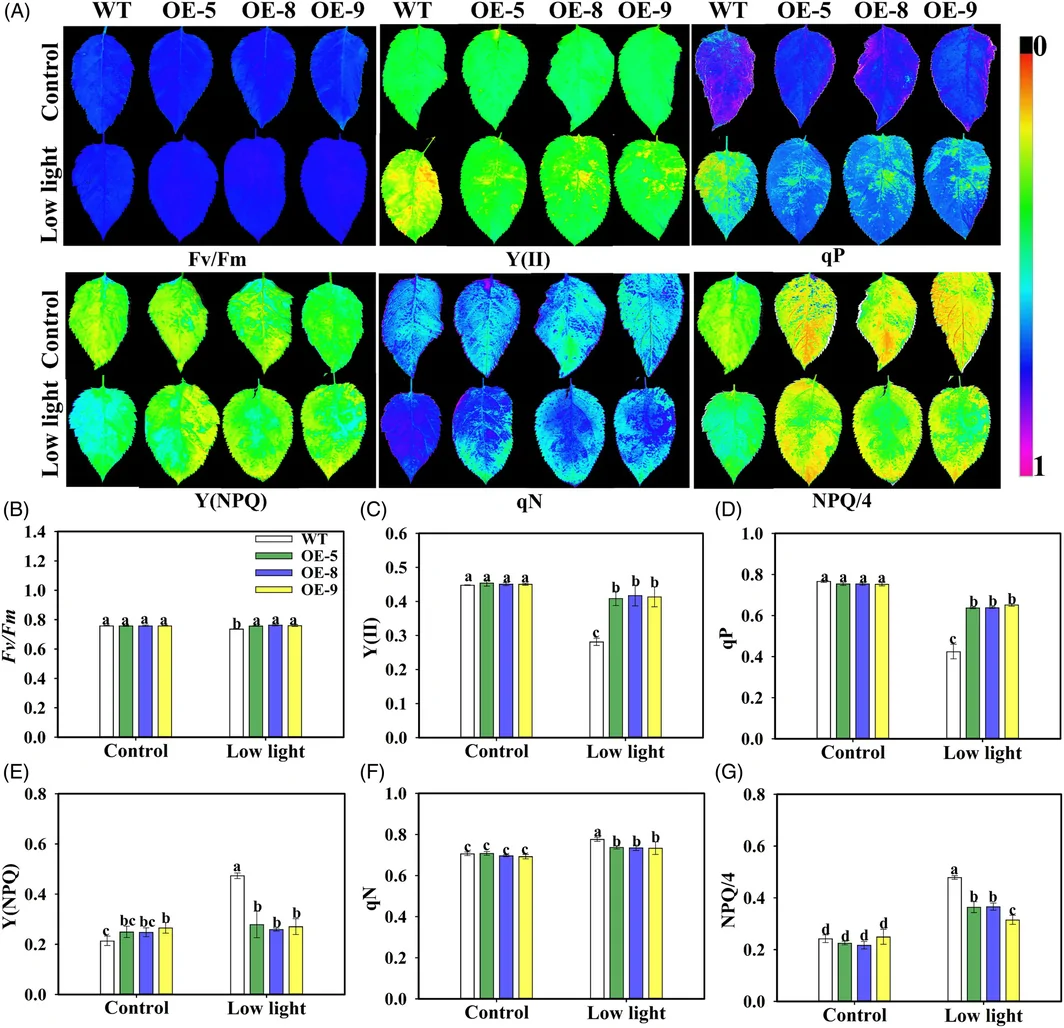
Effects of chlorophyll fluorescence parameters on *MdATG5a*‐OE and WT apple plants under low light. (A) The chlorophyll fluorescence images in WT and *MdATG5a*‐OE transgenic apple plants under low light. (B–G) Values of *Fv/Fm* (B), Y(II) (C), qP (D), Y(NPQ) (E), qN (F), and NPQ/4 (G) in WT and *MdATG5a*‐OE transgenic apple plants under low light. WT, wild type, here, we used ‘GL‐3’ apple (*M. domestic*), which was also used as explants in generating transgenic apple plants; OE‐5/8/9: transgenic apple plants overexpressed *MdATG5a*, which was obtained previously. Data are means ± SD. Different letters indicated that there were significant differences in Tukey's multiple test data (*P* < 0.05).

To further explore the role of *MdATG5a* in apple plants in light‐energy conversion efficiency, we measured the chlorophyll fluorescence of leaves in all genotypes and analyzed relevant parameters (Figure 4). The fluorescence images and values of almost all the parameters varied little between WT and OE plants under control conditions. After low‐light treatment, PSII energy conversion efficiency decreased in all genotypes. Fv/Fm remained nearly unchanged in OE plants before and after low‐light treatments, but it clearly decreased in WT plants after treatment (Figure 4B). Reductions in Y(II) and qP were less pronounced in OE plants compared with WT plants after low‐light treatment (Figure 4C,D). The non‐photochemical quenching indicators Y(NPQ), qN, and NPQ/4 were higher in WT plants than in OE plants under low light (Figure 4E–G).

**Figure 5:**
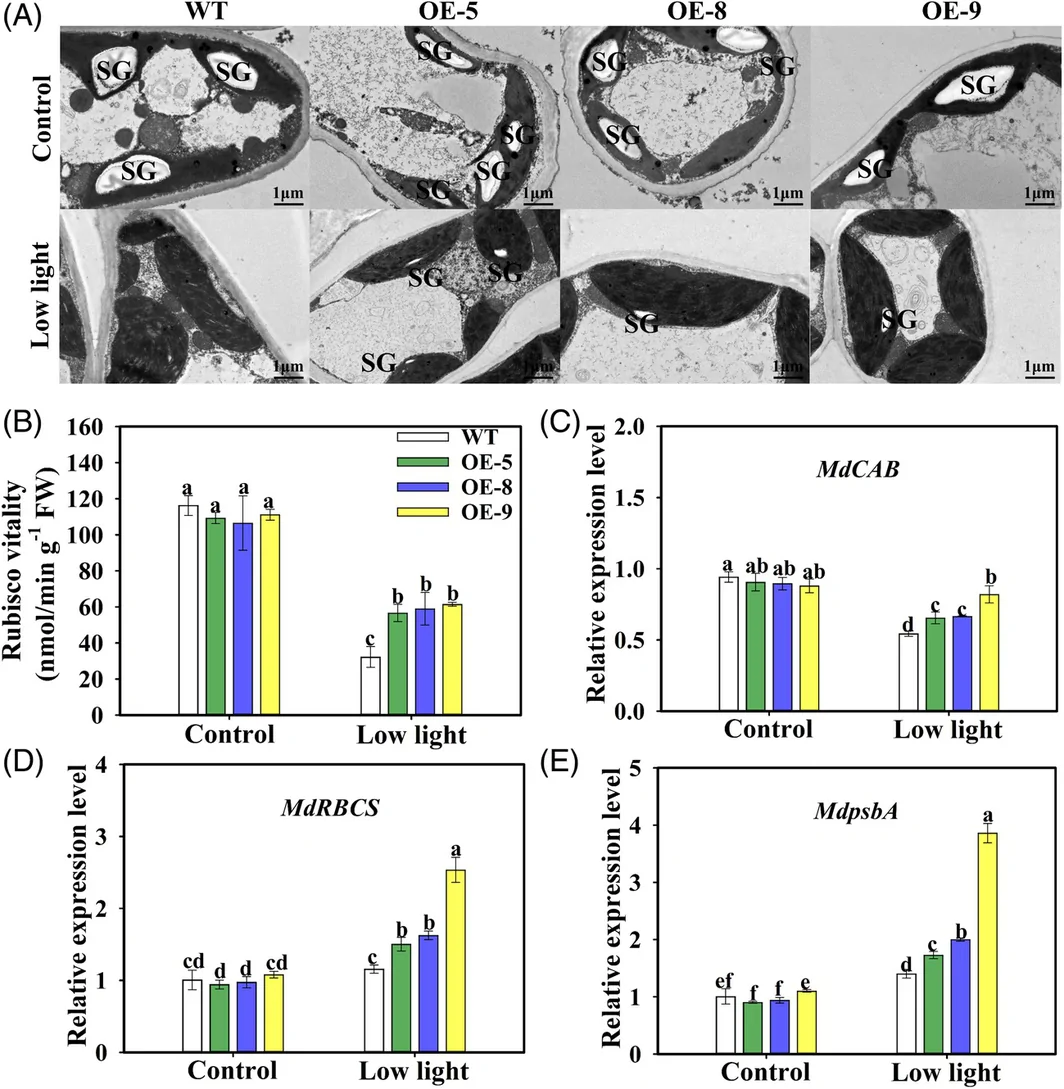
Effects of chloroplast on *MdATG5a*‐OE and WT apple plants after low‐light treatment. (A) Observation of chloroplast morphology of apple leaves by transmission electron microscopy (TEM). SG refers to starch granules. The scale bar = 1 μm. (B) Rubisco vitality. (C–E) The relative expression level involved in chloroplast activity, including *MdCAB* (C), *MdRBCS* (D) and *MdpsbA* (E) in WT and *MdATG5a*‐OE transgenic apple plants under low light. WT, wild type, here, we used ‘GL‐3’ apple (*M. domestic*), which was also used as explants in generating transgenic apple plants; OE‐5/8/9: transgenic apple plants overexpressed *MdATG5a*, which was obtained previously. Data are means ± SD. Different letters indicated that there were significant differences in Tukey's multiple test data (*P* < 0.05).

Chloroplast morphology was normal, and many starch granules were observed in the leaves of all genotypes in the control group. Under low light, the chloroplasts in WT cells were significantly swollen, with sparse grana lamellae and fewer starch grains; in OE plants, chloroplast ultrastructure was less damaged, and small starch grains were present (Figure 5A). Low light affects plant Rubisco activity, resulting in blocked CO_2_ electron transfer (Sun et al., 2014). Rubisco activity decreased more markedly in WT plants than in OE plants under low‐light treatment (Figure 5B). We also detected the expression levels of three genes involved in chloroplast activity (*MdCAB*, *MdRBCS*, and *MdpsbA*). Although the expression of *MdCAB* decreased, while that of *MdRBCS* and MdpsbA increased in response to low‐light stress, their expression levels were consistently higher in OE plants than in WT plants (Figure 5C–E). These findings suggest that *MdATG5a* overexpression in apple plants increases light‐energy conversion efficiency by alleviating damage to the photosynthetic apparatus under low light.

### Effect of *MdATG5a* on nutrient distribution and accumulation in apple plants under low light

**Figure 6:**
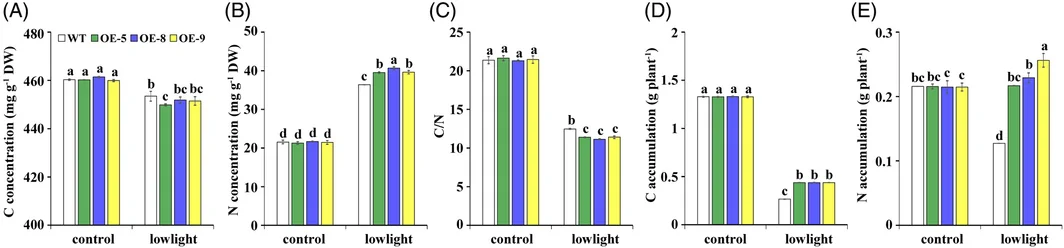
Effects of carbon (C) and nitrogen (N) concentrations and accumulation in the whole plant on *MdATG5a*‐OE and WT apple plants after low‐light treatment. (A–E) Analysis of carbon concentration (A), nitrogen concentration (B), C/N (C), carbon accumulation (D) and nitrogen accumulation (E) of total plants in WT and *MdATG5a*‐OE transgenic apple plants under low light. WT, wild type, here, we used ‘GL‐3’ apple (*M. domestic*), which was also used as explants in generating transgenic apple plants; OE‐5/8/9: transgenic apple plants overexpressed *MdATG5a*, which was obtained previously. Data are means ± SD. Different letters indicated that there were significant differences in Tukey's multiple test data (*P* < 0.05).

We examined the assimilation and distribution of C and N among organs under low‐light stress. Under control conditions, C concentrations in individual tissues and whole plants were similar between WT and OE lines. Low light markedly reduced C concentrations in all tissues. The decrease in C was smaller in roots but more pronounced in stems and leaves in OE plants than in WT plants (Figure S2A). Differences in the whole‐plant C concentration between OE and WT plants were small, except for the OE‐5 line (Figure 6A). Inorganic N in chloroplasts can account for up to 75% of total leaf N (Ghimire et al., 2017) and is therefore closely associated with photosynthesis. In contrast to C, N concentrations increased in all genotypes in response to low light; they were higher in stems and leaves but lower in the roots of OE plants than in WT plants (Figure S2B). Consequently, the whole‐plant N concentration was higher in OE plants than in WT plants under low‐light stress (Figure 6B). The C/N ratio within each organ followed the same trend as the C concentration (Figure S2C) and was lower in whole OE plants than in whole WT plants (Figure 6C).

In addition, whole‐plant C accumulation was significantly reduced by low‐light stress but remained markedly higher in OE plants than in WT plants (Figure 6D). Tissue‐level analysis showed that C accumulation decreased in all genotypes under low light; however, this reduction was more pronounced in WT plants than in OE plants (Table 3), suggesting that OE plants accumulated more carbohydrates than WT plants under low light. The starch and soluble sugar contents were also consistent with this pattern (Figure S3). N accumulation under low light decreased in WT lines but increased in OE plants, especially in OE‐8 and OE‐9 (Figure 6E). Under low light, N accumulation declined in all tissues of WT plants; in OE plants, N accumulation significantly increased in stems and leaves (Table 3). These results indicate that *MdATG5a* overexpression enables apple plants to accumulate C and N under low‐light stress, with higher leaf N contributing to stronger photosynthetic performance.

**Table 3:** Carbon (C) and nitrogen (N) contents in different tissues of WT and *MdATG5a*‐OE plants under low‐light treatment (g plant^−1^)

	C contents in Root	C contents in Stem	C contents in Leaf	N contents in Root	N contents in Stem	N contents in Leaf
Control						
WT	0.429 ± 0.0015a	0.5436 ± 0.0003a	0.3558 ± 0.0029b	0.066 ± 0.0014a	0.0508 ± 0.0019b	0.0989 ± 0.0042c
OE‐5	0.422 ± 0.0009a	0.5453 ± 0.001a	0.3593 ± 0.0045ab	0.0661 ± 0.0005a	0.052 ± 0.0003b	0.0975 ± 0.0036c
OE‐8	0.4226 ± 0.0004a	0.5437 ± 0.0003a	0.3649 ± 0.0035a	0.0652 ± 0.0002a	0.0503 ± 0.0013b	0.0992 ± 0.0083c
OE‐9	0.4244 ± 0.0022a	0.5418 ± 0.0031a	0.3615 ± 0.0022ab	0.0653 ± 0.0005a	0.0498 ± 0.0007b	0.0997 ± 0.0059c
Low light						
WT	0.0507 ± 0.0002c	0.1076 ± 0.0001c	0.1077 ± 0.001d	0.0338 ± 0.0003b	0.0331 ± 0.0001c	0.0604 ± 0.0001d
OE‐5	0.074 ± 0.0003b	0.1661 ± 0.0002b	0.1969 ± 0.0002c	0.0333 ± 0.0002b	0.0545 ± 0.0007a	0.129 ± 0.0007b
OE‐8	0.073 ± 0.0042b	0.1691 ± 0.0001b	0.1952 ± 0.0008c	0.0334 ± 0.0023b	0.0565 ± 0.0009a	0.1393 ± 0.0062b
OE‐9	0.073 ± 0.0001b	0.168 ± 0.0005b	0.1959 ± 0.0002c	0.0336 ± 0.0014b	0.0553 ± 0.0005a	0.1673 ± 0.0114a

*Notes*: Control, plants without any treatment; Low light, plants with low‐light treatment. WT, wild type, here, we used ‘GL‐3’ apple (*M. domestic*), which was also used as explants in generating transgenic apple plants; OE‐5/8/9: transgenic apple plants overexpressed *MdATG5a*, which was obtained previously. Data are means ± SD. Different letters indicated that there were significant differences in Tukey's multiple test data (*P* < 0.05).

### Effect of *MdATG5a* overexpression on metabolites in apple plants under low light

Metabolomics analysis was conducted to further characterize differences in the responses of WT and OE plants to low light. A total of 1290 metabolites were detected and grouped into 20 classes, including 729 in positive ion mode and 561 in negative ion mode (Figure S4A). Flavonoids, amino acids and their derivatives, lipids, organic acids and their derivatives, and carbohydrates and their derivatives together comprised approximately half of the detected metabolites (Figure S4B), but accounted for nearly 90% of total metabolite abundance. Among these, flavonoids showed a pronounced decrease under low light in both WT and OE plants, which likely contributed to the overall reduction in metabolite abundance under low‐light stress (Figure S4C).

**Figure 7:**
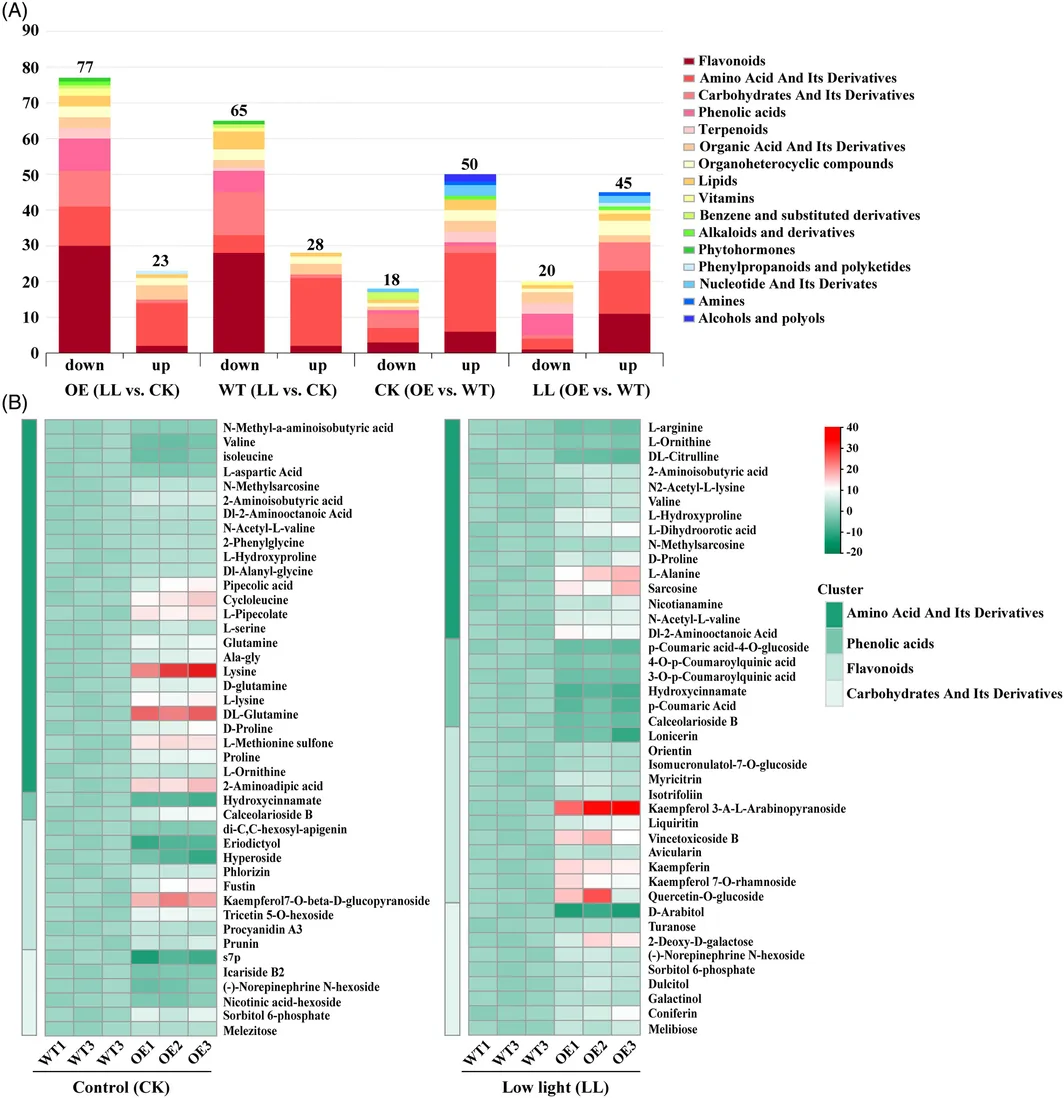
Analysis of DAMs in *MdATG5a*‐OE and WT apple plants after natural and low‐light treatments. (A) Statistical map of the number and type of differential metabolites. (B) The heatmap of differential metabolites in the amino acid and its derivatives, phenolic acids, flavonoids, carbohydrates and its derivatives. CK refers to natural light control group; LL refers to low‐light group. WT, wild type, here, we used ‘GL‐3’ apple (*M. domestic*), which was also used as explants in generating transgenic apple plants; OE: transgenic apple plants overexpressed *MdATG5a*, which was obtained previously.

To identify DAMs, we applied the OPLS‐DA model with VIP ≥1 and *t*‐test *P* < 0.05 as the screening criteria. Relative to the control, low light markedly suppressed metabolism in apple plants, as indicated by the higher number of downregulated DAMs in both WT and OE lines (65 and 77, respectively). Most of these DAMs were annotated as flavonoids, amino acids and their derivatives, carbohydrates and their derivatives, or phenolic acids, with flavonoids alone accounting for nearly half of the downregulated DAMs in both genotypes. In the amino acids and their derivatives category, OE plants exhibited nearly equal numbers of up‐ and downregulated DAMs (12 and 11, respectively) in response to low light, whereas WT plants showed many more upregulated (19) than downregulated (5) DAMs in this group (Figure 7A; Table S2, LL vs. CK).

We further examined DAMs within these four metabolite classes in OE vs. WT comparison groups under both control and low‐light conditions. In these comparisons, most DAMs in the amino acids and their derivatives category were upregulated, regardless of light regime. By contrast, phenolic acids tended to be downregulated, while flavonoids and carbohydrates and their derivatives were generally upregulated under low light compared with the control (Figure 7B). In other words, under low light, OE plants reduced the accumulation of DAMs in phenolic acids, but promoted the accumulation of DAMs in flavonoids and carbohydrates and their derivatives. These metabolic adjustments may facilitate the enhanced tolerance of OE plants to low‐light stress.

### *MdATG5a* ‐mediated autophagic activity is directly associated with the low‐light response in apple plants

**Figure 8:**
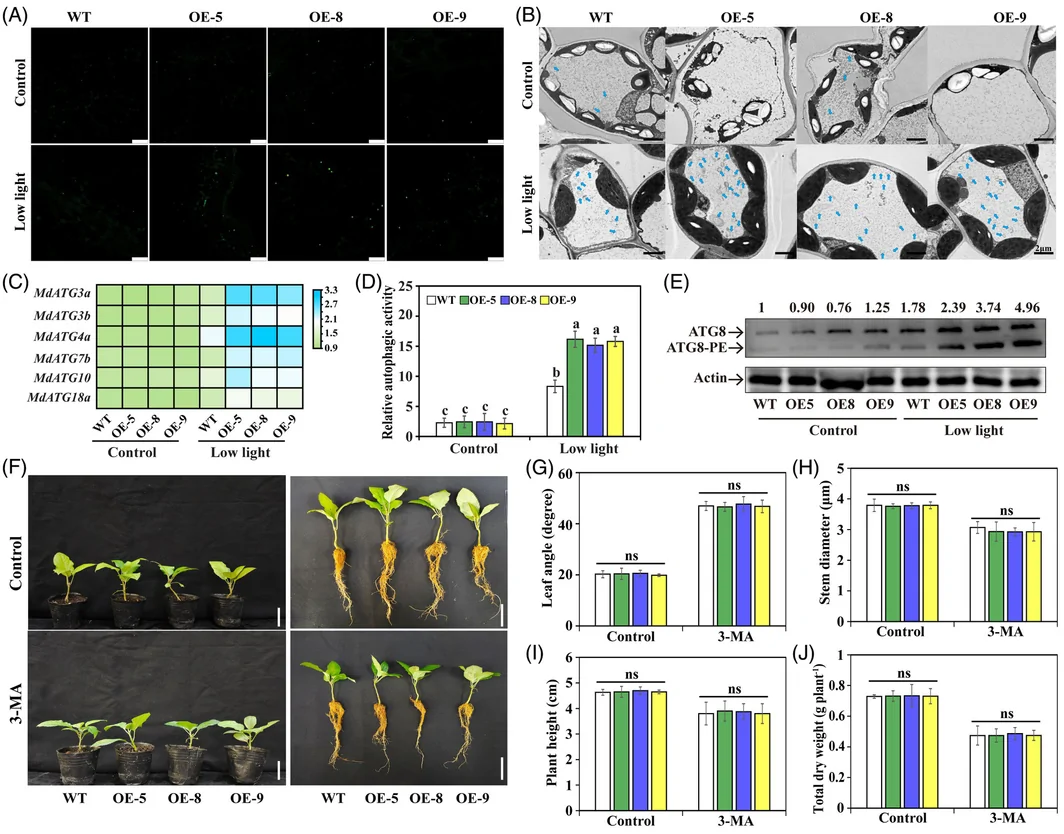
Effects of autophagy on *MdATG5a*‐OE and WT apple plants after low‐light treatment. (A) MDC‐stained autophagosomes in WT plants under control or treatment conditions. The leaves were stained with MDC and visualized by fluorescence confocal microscopy. Bars = 50 um, with more than 10 images analyzed for each treatment. (B) Images of autophagic structures in mesophyll cells of WT and *MdATG5a*‐OE transgenic apple plants under low‐light treatment by using the transmission electron microscope (TEM). The scale bar = 2 μm. At least 10 different cells were used to quantify the autophagic structures. Autophagic bodies are indicated by blue arrows. (C) The expression patterns of apple autophagy‐related genes (*MdATG3a*; *MdATG3b*; *MdATG4a*; *MdATG7b*; *MdATG10*; *MdATG18a*) in WT and *MdATG5a*‐OE transgenic apple plants under low‐light treatment. (D) The relative autophagic activity of WT and *MdATG5a*‐OE transgenic apple plants. (E) ATG8 protein levels in the WT and MdATG5a‐OE plants in control or low‐light treatment. ATG8 (non‐lipidated form) and ATG8‐PE (lipidated form) are indicated on the left. The ratio of ATG8‐PE to ATG8 reflects the level of autophagy accumulation, with the ratio in WT plants under control conditions set to 1.00. Actin was used as an internal control. The experiment was repeated 3 times with similar results. Leaves were collected at 30 days after low‐light treatment. (F) Phenotypes of WT and *MdATG5a*‐OE transgenic apple plants after spraying autophagy inhibitor 3‐MA on leaves under low light. The scale bar = 5 cm. (G–J) Values of the leaf angle (G), plant height (H), stem diameter (I) and total dry weight (J) in WT and *MdATG5a*‐OE transgenic apple plants under low light. WT, wild type, here, we used ‘GL‐3’ apple (*M. domestic*), which was also used as explants in generating transgenic apple plants; OE‐5/8/9: Transgenic apple plants overexpressed *MdATG5a*, which was obtained previously. Data are means ± SD. Different letters indicated that there were significant differences in Tukey's multiple test data (*P* < 0.05). ns, no significant difference.

We analyzed the expression patterns of several key *MdATG* genes (*MdATG3a*, *MdATG3b*, *MdATG4a*, *MdATG7b*, *MdATG10*, and *MdATG18a*) by qRT‐PCR. Under control conditions, their transcript levels did not differ significantly among genotypes. By contrast, low‐light stress induced the expression of these genes in every genotype, with OE plants showing significantly higher transcript levels than WT plants (Figure 8C). MDC staining showed much more punctate fluorescence signals in OE apple leaf cells than in WT under low light (Figure 8A). The transmission electron microscope (TEM) analyses also showed that a greater number of autophagosomes in OE leaf cells (Figure 8B), the number of autophagosomes was 1.94‐, 1.82‐, and 1.90‐fold higher in OE‐5, OE‐8, and OE‐9 plants, respectively, than in WT plants (Figure 8D). In addition, we detected the abundances of ATG8 and ATG8‐PE in apple plants using anti‐ATG8 antibody. Notably, ATG8‐PE bands were obviously detected in the low‐light treated plants, and the ratio of ATG8‐PE to ATG8 was notably higher in OE apple plants compared with WT plants after low‐light treatment (Figure 8E). Together, these results indicate that *MdATG5a* overexpression further enhances autophagic activity in apple plants under low‐light conditions.

To further test whether *MdATG5a*‐mediated autophagy is functionally involved in low‐light responses, we treated both OE and WT plants with 3‐MA, an autophagy inhibitor, under low light. Photosynthetic response curves were first determined using seedlings grown in an artificial climate chamber (Figure S5). A light intensity of 3500 lux (50 μmol m^−2^ s^−1^) was used as the low‐light treatment and 10 000 lux (120 μmol m^−2^ s^−1^) as the control in the chamber. Under low light, differences in plant height, stem thickness, total fresh weight, and total dry weight between WT and OE plants were consistent with those observed under outdoor conditions (Figure S6). Leaf angles increased in all genotypes, and the change in leaf angle was particularly pronounced in WT plants (Figure S6B). However, following 3‐MA application, the differences between WT and OE plants under low light disappeared for all measured traits (Figure 8F–J), supporting a direct role for *MdATG5a*‐mediated autophagic activity in the low‐light response of apple plants.

### MdPIF7 directly binds to the promoter of *MdATG5a* and activates its expression

**Figure 9:**
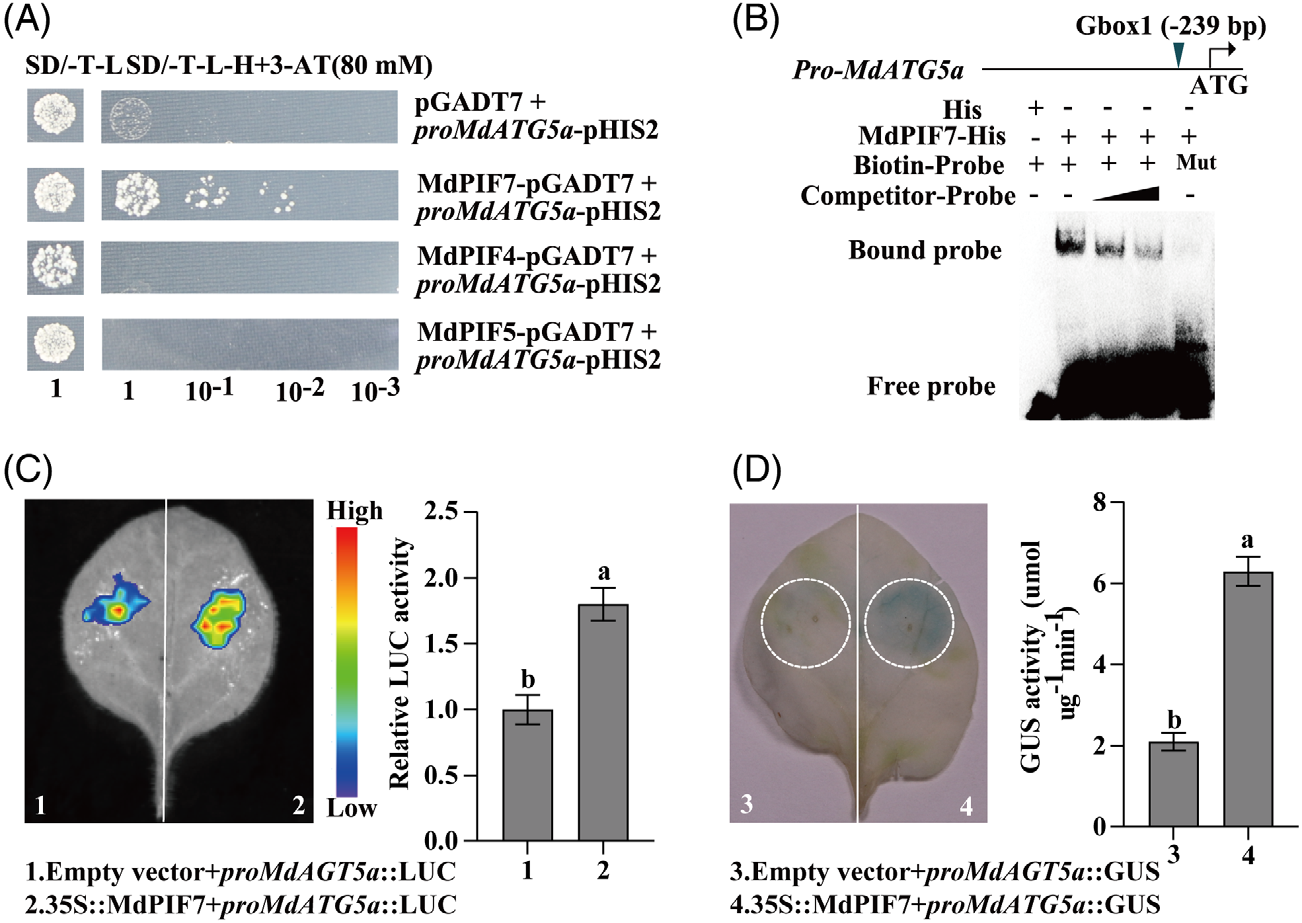
MdPIF7 binds to the *MdATG5a* promoter and activates its transcription. (A) Y1H assays between the interaction of MdPIF7 and the promoter of *MdATG5a*. –T/−L, −Trp‐Leu; −T/−L/‐H, −Trp‐Leu‐His; 3‐AT, 3‐amino‐1,2,4‐triazole; Y1H, yeast one‐hybrid. (B) Competing EMSAs of the interaction between MdPIF7 and 5′‐biotin labeled probes. Competitor‐Probe, without biotin labeling. Mut, mutant probes in which the 5′‐CACGTA‐3′motifs were replaced by 5′‐AAGGAA‐3′. EMSA, electrophoretic mobility shift assay. (C) Dual‐luciferase reporter· assay to analyze the activation effect of MdPIF7 on the *MdATG5a* promoter in N. benthamiana leaves. (D) The staining and activities of GUS between the interaction of MdPIF7 and the promoters of *MdATG5a*. Darker blue indicates stronger GUS activity. β‐glucuronidase (GUS) assay and dual‐luciferase reporter assay were used to analyze the activation effect of MdPIF7 on the *MdATG5a* promoter in Nicotiana benthamiana leaves. Data are means ± SD. Different letters indicated that there were significant differences in Tukey's multiple test data (*P* < 0.05).

Phytochrome‐interacting factors (PIFs), a subfamily of basic helix–loop–helix (bHLH) transcription factors, are key regulators of light signaling in apple (Zheng *et al*., 2020). PIF4/5/7 have been shown to mediate shade avoidance responses, enabling plants to sustain growth and adaptability under low‐light conditions (de Wit *et al*., 2016). We performed binding assays to determine whether MdPIF4/5/7 directly regulate *MdATG5a* expression in response to low light. Y1H assays showed that only yeast cells co‐expressing MdPIF7 and *proMdATG5a* grew on SD/−Trp/−Leu/‐His medium containing 80 mM 3‐AT, whereas cells harboring pGADT7‐MdPIF4 or pGADT7‐MdPIF5 together with pHIS2‐*proMdATG5a* failed to grow under the same conditions (Figure 9A).

To further confirm that MdPIF7 binds to *proMdATG5a*, we performed EMSAs. MdPIF7 directly bound to the G‐box1 (−239 bp) motif of *proMdATG5a*, but not to G‐box2 (−492 bp) (Figure S7). A competitive EMSA showed that this binding to G‐box1 was progressively weakened by increasing amounts of unlabeled specific competitor, and mutation of the core nucleotides within G‐box1 completely abolished MdPIF7 binding (Figure 9B). Dual‐LUC and GUS assays in *N. benthamiana* leaves further demonstrated that co‐expression of 35S::MdPIF7 significantly enhanced *proMdATG5a*‐driven luminescence (Figure 9C), as well as the blue coloration intensity and quantitative fluorometric GUS activity (Figure 9D). In addition, we performed qRT‐PCR analysis and found that the expression level of *MdPIF7* was induced by low‐light treatment (Figure S8A). And the in vivo Dual‐LUC assay showed that the transcriptional activation of MdPIF7 on the *MdATG5a* promoter was further strengthened by the low‐light treatment (Figure S8B). Together, these results demonstrate that MdPIF7 directly binds to the *MdATG5a* promoter and activates its expression under low‐light stress.

## DISCUSSION

### *MdATG5a* overexpression enhanced photosynthesis in apple plants under low light

Light is a critical factor affecting plant photomorphogenesis, growth, and development. Insufficient light reduces carbohydrate synthesis in plants, which, in turn, promotes autophagy to help maintain intracellular homeostasis. Previous studies have shown that overexpression of the autophagy‐related gene *MdATG5a* positively regulates N use efficiency under low‐N conditions (Jia et al., 2022). Here, we found that *MdATG5a*‐mediated autophagic activity positively regulates the low‐light response of apple plants, and that inhibition of autophagy abolishes the significant differences between WT and OE plants.

Apple is a sun‐loving species, and low‐light stress reduces its photosynthetic capacity and strongly affects leaf physiology and chloroplast ultrastructure (Schramma et al., 2023). SLW is a key parameter reflecting leaf structural properties and is closely related to the light environment and leaf photosynthesis (Meir et al., 2002). In plants growing at high density, in understory habitats, or under low‐light stress, leaves with lower SLW tend to be thinner, which often improves light capture and enhances photosynthetic capacity (Niinemets et al., 2015; Yao et al., 2015). Under low light, *MdATG5a*‐OE plants exhibited lower SLW and larger, thinner leaves than WT plants, suggesting that OE plants may capture more light energy under these conditions. When photosynthetically active radiation is reduced, plants can compensate by increasing light‐use efficiency (Charbonnier et al., 2017). For example, densely planted maize improves light utilization by increasing electron transport efficiency and shows higher AQE, LCP, and Rd (Zheng et al., 2022). In our study, low light decreased LSP, LCP, Rd, and Amax and increased AQE in all genotypes. Moreover, OE plants had lower LSP, LCP, and Rd but higher Amax and AQE than WT plants. Therefore, *MdATG5a* appears to facilitate adaptation to low light by decreasing LCP to enhance light‐use efficiency, reducing Rd to lower the consumption of organic matter, and decreasing LSP so that maximum Pn is achieved at lower light intensity.

Moreover, Pn, Gs, and light utilization efficiency per unit incident light (Pn/PARi) were higher in OE plants than in WT plants under low light, accompanied by lower Ci, indicating that the decrease in Pn was not caused by stomatal limitation (Shi *et al*., 2022). Instead, it is likely associated with reduced PSII activity and damage to photosynthetic organs. Low‐light stress primarily impairs PSII function in plants (Sharma et al., 2012). In our study, OE plants exhibited higher PSII energy conversion efficiency than WT plants under low light, highlighting a protective role of autophagy in maintaining PSII function.

Previous studies have shown that shading reduces crop photosynthetic capacity and alters the structure of photosynthetic organs (Königer et al., 2008; Long et al., 2022; Moisan et al., 2006; Zhang, Chen, et al., 2022), and chloroplast integrity is crucial for normal plant physiological function (Huo et al., 2020). Here, we found that OE plants had fewer damaged chloroplasts and displayed higher expression levels of *MdCAB*, *MdRBCS*, and *MdpsbA* under low light. Under stress conditions, plants promote the degradation of damaged chloroplast proteins (Wan et al., 2023; Zhang & Ling, 2024), and activation of autophagy can alleviate the adverse effects caused by the absence of chloroplast vesicles (CVs) during prolonged darkness (Barros et al., 2023). Consistent with this, we observed stronger autophagic activity in OE plants under low light. These findings suggest that *MdATG5a*‐mediated autophagy enhances photosynthesis by mitigating damage to the photosynthetic apparatus of apple under low‐light conditions.

### *MdATG5a* overexpression altered C and N allocation in apple plants under low light

C and N are essential elements for plant growth and development. Their assimilation capacity and distribution within tissues not only influence organ development but also reflect the adaptability of plants to their environment. N metabolism depends on CO_2_ assimilation (C metabolism) to provide the necessary C skeletons and energy, while C metabolism, in turn, requires N metabolism to supply enzyme proteins and photosynthetic pigments (Xu et al., 2023). Leaf N is predominantly localized in chloroplasts involved in photosynthesis, and higher leaf N levels generally enhance photosynthetic efficiency (Ghimire et al., 2017; Wei et al., 2022). Under low light, we found that *MdATG5a* overexpression significantly increased the leaf N content and total N accumulation in apple plants, which would enable OE plants to synthesize more light‐harvesting proteins and thereby improve their low‐light tolerance (Wu et al., 2022; Zhuo et al., 2024). Rubisco is a key photosynthetic enzyme, and its activity strongly influences photosynthetic capacity before the saturating light intensity is reached (Sugiura & Tateno, 2011). In this study, low light reduced Rubisco activity in all genotypes; however, Rubisco activity remained higher in OE plants than in WT plants, likely benefiting from their elevated leaf N accumulation.

High light typically enhances photosynthesis and increases the demand for water and minerals, which promotes greater C allocation to the roots and the development of a more extensive root system (De Lara‐Del Rey & Pérez‐Fernández, 2023; Oliveira et al., 2019). By contrast, low light reduces carbohydrate synthesis and promotes N metabolism, leading to greater biomass allocation to aboveground organs and a decreased root:shoot ratio (Wu et al., 2022). In line with these findings, we observed higher leaf biomass allocation but lower root allocation and a reduced root:shoot ratio in OE plants compared with WT plants. Nevertheless, the C content and total C accumulation were still higher in the roots of OE plants than in those of WT plants under low light. This indicates that although low light generally promotes shoot growth and suppresses root development, *MdATG5a* further amplifies the beneficial changes while mitigating detrimental ones. As a result, OE plants develop more robust leaf and root systems compared with WT plants under low‐light conditions.

The differential allocation in C and N also directly affects their metabolism, which represents the two central primary metabolic pathways for plant growth and development, and they exert far‐reaching effects on the maintenance of plant metabolic homeostasis. The metabolome analysis revealed dramatic remodeling of core DAMs in *MdATG5a*‐OE plants, particularly metabolites belonging to carbohydrate, sugar alcohol, and amino acid derivative categories. The accumulation of metabolites in the sugars, sugar alcohols, and TCA cycle groups paralleled the enhanced carbohydrate reserves and photosynthetic performance of *MdATG5a*‐OE plants. Additionally, the shifted amino acid derivatives aligned with increased leaf N content, higher activities of N assimilation enzymes, and faster Rubisco turnover, synergistically boosting whole‐plant C and N sequestration. Collectively, these metabolic shifts reflect the coordinated reconfiguration of C assimilation, N remobilization, and central energy metabolism under low‐light conditions.

### *MdATG5a* relies on MdPIF7 to regulate low‐light adaptation in apple

Light is an important environmental signal that is perceived by photoreceptors such as phytochromes and cryptochromes, which detect changes in light quality, intensity, and photoperiod. Light perception activates downstream transcription factors, including PIFs, HY5, and CRYs, to coordinate plant growth, development, and stress adaptation (Chen et al., 2023; Han et al., 2023; White, 2022). Notably, key light‐signaling transcription factors, particularly PIFs and HY5, have been shown to bidirectionally regulate autophagy gene expression in a manner highly dependent on environmental conditions. Under favorable light conditions, HY5 represses the transcription of *ATGs* and maintains low basal autophagy flux (Yang et al., 2020). HY5 can itself be degraded by autophagy, and its nuclear stability contributes to the suppression of *ATG* expression (Jiang et al., 2025).

PIF7 is a central regulator of the shade response. As a transcription factor, it indirectly senses shade through phyB‐dependent and phyA‐mediated pathways (Casal & Fankhauser, 2023; Yang et al., 2017). PIF7 coordinately regulates downstream shade signaling via the auxin pathway and chromatin remodeling (Xu et al., 2024). The PIF7‐mediated shade signaling mechanism is well established in model plants such as *Arabidopsis* and tobacco, and its function is highly conserved in higher plants (Cheng et al., 2024; Jiang et al., 2019; Patitaki et al., 2022; Yang et al., 2023; Zou et al., 2020). In this study, we provide the first evidence that PIF7 directly activates the transcriptional activity of the autophagy gene *MdATG5a* in apple under low‐light stress. MdPIF7 directly binds to the G‐box1 element located at −239 bp in the *MdATG5a* promoter under low‐light conditions, as demonstrated by Y1H, EMSA, and dual‐luciferase reporter assays. In contrast to the inhibitory effect of the HY5–HDA9 complex on *ATG5* and *ATG8e*, MdPIF7 promotes the expression of *MdATG5a* in response to low light, revealing a bidirectional regulatory mechanism by which light governs autophagy. In essence, autophagy is suppressed under favorable light to promote growth but activated under stress conditions, such as low light, to maintain cellular homeostasis.

MdPIF7 mediated transcriptional activation of *MdATG5a* further coordinates whole‐plant C and N partitioning to reshape nutrient distribution and optimize photosynthetic acclimation under low‐light conditions as mentioned above. For instance, it remobilized N from root storage pools and senescent intracellular proteins toward functional leaves (Figure 6B,E; Jia et al., 2022). And it amplified the shade‐triggered biomass allocation shift toward leaves (Figure 1F–J). These changes further cascade down to metabolic processes. The metabolic stability of flavonoids and carbohydrates in *MdATG5a*‐OE plants under low light can be largely attributed to enhanced *MdATG5a*‐mediated autophagic activity. When treated, the *MdATG5a*‐OE plants with 3‐MA to pharmacologically inhibit the autophagy activity, the growth and metabolic advantages of *MdATG5a*‐OE plants were completely abolished. So, MdPIF7 mediated activation of *MdATG5a* elevated autophagy, thus preventing excessive depletion of flavonoids and non‐structural carbohydrates, thereby buffering plants against low‐light‐induced metabolic perturbation.

In addition, the regulation of autophagy genes by light‐signaling transcription factors is highly context‐dependent, not only on light conditions but also on other environmental cues. For example, PIF4 can be activated by heat stress, which then induces *ATG* expression and promotes thermomorphogenesis partly through the selective degradation of the circadian clock protein TOC1 (Li et al., 2025; Wainstock, 2012). These regulatory events in different stress responses share common signaling nodes and mechanisms, suggesting the existence of partially overlapping transcriptional networks that integrate diverse environmental signals. This provides a theoretical framework for understanding the cross‐environmental regulation of *ATG* genes in plants.

In summary, our study demonstrates that *MdATG5a* is directly regulated by MdPIF7 in response to low‐light stress, leading to high *MdATG5a* expression and elevated autophagy activity, which are directly linked to the low‐light response of apple plants (Figure S9). *MdATG5a* improves photosynthetic performance mainly through four mechanisms: first, expanding leaf area to increase light‐energy capture; second, increasing the leaf N content to promote the synthesis and turnover of photosynthetic enzyme proteins and pigments; third, lowering LCP, LSP, and Rd to maximize Pn while reducing C consumption; and fourth, mitigating damage to chloroplasts and PSII to enhance light‐energy conversion efficiency in apple plants. Together, these *MdATG5a*‐mediated changes enable apple plants to accumulate more biomass and exhibit superior growth under low‐light environments. That might help us improve light‐use efficiency of apple plants by regulating autophagy activity, and aid the breeding of low‐light‐tolerant germplasm for orchard systems with suboptimal light conditions.

## MATERIALS AND METHODS

### Plant materials and treatments

Tissue‐cultured ‘GL‐3’ (*Malus domestica* ‘Royal Gala’) was used as the WT in this study. *MdATG5a*‐OE apple lines were generated previously (Jia et al., 2022). Both OE and WT were cultured as described by Xiang et al. (2024). Briefly, after 35 days on rooting media, WT and OE lines were first transferred and cultured in a plant growth chamber under a 16 h/8 h light/dark photoperiod (120 μmol photons m^−2^ s^−1^) at 24°C for 4 weeks. They were then transplanted and acclimated for 2 weeks in the greenhouse and subsequently outdoors at the agricultural experimental station of Northwest A&F University.

Apple plants of similar size were subjected to low‐light treatment. Plants grown under natural sunlight served as the control (Control). Apple plants enclosed on all sides with a 12‐pin shade net comprised the low‐light treatment group (Low Light), wherein the light intensity was reduced to approximately 10% of natural sunlight (Figure S1A). The experimental period lasted 60 days (May 24 to July 24, 2023), during which daily weather conditions were recorded (Figure S1B). Sampling and data collection were conducted at various time points during the treatments according to different measurements, and biomass was measured on 0 and 60 days.

To verify whether autophagy responds to low light, an additional experiment was conducted in which we applied the autophagy inhibitor 3‐methyladenine (3‐MA) as a foliar spray to both WT and OE apple plants under low‐light conditions in an artificial climate chamber. The photosynthesis response curve of apple seedlings under different light environments was determined using the CIRAS‐3 system (CIRAS‐3, Amesbury, MA, USA). The light intensity was set to 120, 100, 85, 60, 50, 30, and 0 μmol m^−2^ s^−1^, corresponding to 10 000, 8000, 6000, 4000, 3500, 2000, and 0 lux, respectively. The measurements were fitted using a non‐rectangular hyperbolic model to obtain 20 μmol m^−2^ s^−1^ as the light compensation point for apple seedlings. Specifically, 10 000 lux (120 μmol m^−2^ s^−1^) was used as the control light intensity and 3500 lux (50 μmol m^−2^ s^−1^) as the low‐light intensity. The additional 3‐MA treatment was performed under the same low‐light conditions, with leaves sprayed with 10 mM 3‐MA every 7 days, a concentration approximately twice that used in tobacco‐injected experiments (Che et al., 2022). Forty plants in each group were treated, and phenotypes and biomass were recorded at 50 days.

### RNA extraction and quantitative real‐time polymerase chain reaction (qRT‐PCR) analysis

Total RNA was extracted using the Wolact Plant RNA Isolation Kit (Wolact, Hong Kong, China), and first‐strand cDNA was synthesized using the Thermo Revert Aid First Strand cDNA Synthesis Kit (Thermo Scientific, Waltham, MA, USA) with 1 μg of total RNA. qPCR analysis was performed as previously described by Xiang et al. (2024). *MdMDH* was used as the internal reference gene. The primer sequences for expression analysis are provided in Table S1.

### Determination of growth parameters

Ten plants from each line were collected on day 0 and day 60 of the outdoor low‐light treatment to determine growth parameters. The fresh weight (FW), dry weight (DW), relative growth rate (RGR), and root: shoot ratio were calculated according to Xiang et al. (2024). The root/stem/leaf biomass ratio (RMR/SMR/LMR, %) was calculated according to Komiyama et al. (2000). Leaf length, leaf width, and leaf area were measured using a portable leaf area meter. Leaf angle was measured as described by Zhang et al. (2023). Specific leaf weight (SLW) was calculated as previously described (Anitha et al., 2007). Detailed information can be found in the supplemental files.

### Measurements of photosynthesis and chlorophyll fluorescence parameters

During the low‐light treatment, gas exchange parameters were measured at 7:00–9:00 am on a sunny day using a CIRAS‐3 portable photosynthesis system. The fourth to sixth mature leaves from the apex to the stem were used to measure gas exchange parameters. Light utilization efficiency per unit of light intensity (LUE) was determined as described previously (Zheng et al., 2022): LUE = Pn / PARi, where Pn is the net photosynthetic rate, and PARi is the real‐time unit of photosynthetically active radiation. Chlorophyll fluorescence was measured using an IMAGING‐PAM system (Walz, Effeltrich, Germany) after 20 min of dark adaptation of leaves at the same location.

The light gradients were set at 2000, 1700, 1400, 1200, 1000, 800, 600, 400, 300, 200, 150, 100, 50, and 0 μmol m^−2^ s^−1^, and the light‐response curves were measured. The non‐rectangular hyperbolic model was used to obtain the light‐response curve fitting parameters, including AQE, maximum net photosynthetic rate (Amax), LCP, Rd, and LSP. Among these parameters, LSP is calculated as follows (Zheng et al., 2022): Photo = AQE × PAR – Rd. When Photo = Amax, PAR is LSP.

Rubisco activity was determined using the corresponding kits (Suzhou Griseo Biotechnology Co., Ltd., Suzhou, China) according to the manufacturer's instructions.

### Determination of C and N

Dried samples of roots, stems, leaves, and whole plants were ground into powder. Each sample was weighed (100 mg) and placed in a fully automated C and N analyzer (Skalar, Netherlands) to determine the C and N content using the high‐temperature combustion method. The C and N accumulation in each organ and in the whole plant (g) = dry weight (g) × C/N content (mg g^−1^).

Glutamine synthetase (GS) activity, nitrite reductase (NiR) activity, Fd‐glutamate synthetase (Fd‐GOGAT) activity, starch content, and soluble sugar content were determined using corresponding kits (Suzhou Comin Biotechnology Co., Ltd., Suzhou, China) according to the manufacturer's instructions.

### Morphological and structural analyses of the leaves

Four μm thick paraffin sections were made for the mature leaves at the same position excised from each apple line, which were then stained with toluidine blue O for structure analysis, following Zhou et al. (2019). In addition, the sampled leaves were cut into small pieces (0.5 cm × 0.5 cm) and fixed in a glutaraldehyde solution. After fixing, the JEOL‐1230 transmission electron microscope (TEM, Hitachi, Japan) was used to observe leaf chloroplast and autophagosome structures as previously described by Xiang et al. (2024). Autophagy activity was assessed by counting autophagosomes at 2500× magnification under the TEM, and at least 10 different cells were counted for each sample.

### MDC staining

The leaves were cut into small squares measuring ∼3 mm × 3 mm, immersed in a solution of 100 μM MDC dye (Beyotime, C3018S), subjected to vacuum treatment, stained in the dark for 30 min, washed 3 times with 1 × PBS buffer (Solarbio, P1020), and then observed under a confocal microscope (TCS SP8 SR; Leica) for MDC staining to identify autophagosomes. Details on these procedures are provided in Jia et al. (2021).

### Immunoblotting analysis

Protein extraction was carried out following a previously described method (Cheng et al., 2026). For ATG8 determination, the extracted proteins were then separated using 15% SDS‐PAGE with 6 M urea. After separation, these proteins were transferred onto nitrocellulose membranes, which were blocked with a solution of 5% skim milk in TBST buffer (composed of 20 mM Tris at pH 7.5, 150 mM NaCl, and 0.1% Tween 20) at room temperature. The primary antibody was anti‐ATG8 polyclonal antibody (Agrisera AS142769), and the secondary antibody was anti‐rabbit. The membrane was incubated with this antibody. The complexes formed after immunoblotting were made visible using ECL kits (Abcam, ab133409).

### Metabolome analysis

The metabolome analysis of plant leaves was performed following Ye et al. (2025). Briefly, after extracting with an 80% methanol aqueous solution, 20 μL of supernatant was subsequently injected into the LC–MS system for analysis. Quality control (QC) samples were prepared by mixing all sample extracts. The experimental samples were detected using multiple reaction monitoring (MRM) mode, and chromatographic peak integration and correction were performed using SCIEX OSV1.4 software. The variable importance in projection (VIP) scores from the orthogonal partial least squares discriminant analysis (OPLS‐DA) model were used to rank differentially accumulated metabolites (DAMs) between groups. Metabolites with VIP ≥1 and *t*‐test *P* < 0.05 were designated as significant DAMs.

### Assays of the MdPIF7‐mediated regulation of *MdATG5a* expression

Yeast one‐hybrid (Y1H) assay, electrophoretic mobility shift assay (EMSA), dual‐luciferase reporter (Dual‐LUC) assay, and GUS assay were performed to verify whether MdPIFs can bind to the promoter of *MdATG5a* (*proMdATG5a*) and regulate its expression.

For the Y1H, The *Nde* I and *Bam*H I restriction enzymes were used to cleave the pGADT7 vector, and the coding sequences (CDSs) of *MdPIF4*, *MdPIF5*, and *MdPIF7* were cloned into it using homologous ligation. The *EcoR* I and *Sac* I restriction enzymes were used to cleave the pHIS2 vector, and the promoter sequences of *MdATG5a* were cloned into it using homologous ligation. These recombinant plasmids were co‐transformed into the yeast strain Y187 and plated on ‐Trp/‐Leu/‐His medium containing different concentrations of 3‐amino‐1,2,4‐triazole (3‐AT). An empty pGADT7 vector was used as a control. The primers used are listed in Table S1.

For the EMSA, The *Bam*H I and *EcoR* I restriction enzymes were used to cleave the pGEX‐4 T‐1 vector. The CDS of *MdPIF7* was cloned into the pGEX‐4 T‐1 vector and expressed in *E. coli* BL21 (DE3) to obtain the GST‐MdPIF7 fusion protein. The EMSA was performed using a LightShift Chemiluminescent EMSA kit according to the manufacturer's instructions (Thermo Fisher Scientific). All probes were synthesized by Sangon Biotech Co., Ltd. (Shanghai, China), with unlabeled probes serving as competitors. The primers used are listed in Table S1.

In the Dual‐LUC assay, the *Bam*H I and *Hind* III restriction enzymes were used to cleave the pGreenII 62‐SK vector, and the CDS regions of *MdPIF7* was cloned into it to obtain the effectors using homologous ligation. The *SaI* I and *Bam*H I restriction enzymes were used to cleave the pGreenII 0800‐LUC vector, and the promoter sequences of *MdATG5a* were cloned into it to form the pro*MdATG5a*::LUC reporters using homologous ligation. These recombinant plasmids were individually transformed into *Agrobacterium tumefaciens* GV3101. The *Agrobacterium* cultures were grown overnight at 28°C, collected by centrifugation, and resuspended in infiltration buffer (10 mM MgCl_2_, 10 mM MES, 150 μM acetosyringone) to an OD_600_ of 0.6–0.8. The recombinant strains were co‐infiltrated into *Nicotiana benthamiana* leaves. Luminescence was detected using an in vivo imaging system (PlantView100; Guangzhou Biolight Biotechnology Co., Ltd., Guangzhou, China). The primers used are listed in Table S1.

In GUS assays, The *Kpn* I and *Bam*H I restriction enzymes were used to cleave the pCambia2300‐GFP vector, and the CDS regions of *MdPIF7* was cloned into it to form 35S::MdPIF7 using homologous ligation. The *BamH* I and *EcoR* I restriction enzymes were used to cleave the pCambia0390‐GUS vector, and the promoter sequences of *MdATG5a* were cloned into it to form *proMdATG5a*::GUS using homologous ligation. The transformation of GV3101, and the infiltration of *Nicotiana benthamiana* leaves were operated as described in the Dual‐LUC assay. Two days later, the GUS activity was measured using a GUS enzyme activity kit (SL7161) according to the manufacturer's instructions (Coolaber, Beijing, China). A multifunctional enzyme‐linked immunosorbent assay (Victor Nivo, PerkinElmer LLC Co., Ltd., USA) was used to measure the fluorescence values. The emission–excitation wavelength was set to 365 nm. Three biological replicates were used to quantify GUS activity. The primers used are listed in Table S1.

### Statistical analysis

Each experimental setup included three biological replicates. Values are presented as the mean of three independent biological replicates, and error bars represent the standard deviation of the three replicates. The data were statistically analyzed by one‐way ANOVA. Tukey's multiple range test was used to determine significant differences between means (*P* < 0.05) using SPSS 25.0 software (IBM Corp., Armonk, NY, USA).

## Author Contributions

XG and FM conceived and designed the experiments. WX and YF performed the research. YL, QZ, and ZL analyzed the data. WX, YF, and XG wrote the manuscript; XG and FM provided the financial support.

## Competing Interests

None declared.

## Supporting information


**Figure S1.** Recordings of photosynthetic photon flux density (PPFD) and meteorology during low‐light treatment. (A) Measurement of PPFD in natural light control group and low‐light treatment group. (B) 2023 Meteorological data of Yangling from May 24 to July 24.

**Figure S2.** Effects of carbon (C) and nitrogen (N) concentrations in different tissues on *MdATG5a*‐OE and WT apple plants after low‐light treatment. (A–C) Analysis of carbon concentration (A), nitrogen concentration (B), C/N (C) of different tissues in WT and *MdATG5a*‐OE transgenic apple plants under low light. WT, wild type, here, we used ‘GL‐3’ apple (*M. domestic*), which was also used as explants in generating transgenic apple plants; OE‐5/8/9: transgenic apple plants overexpressed *MdATG5a*, which was obtained previously. Data are means ± SD. Different letters indicated that there were significant differences in Tukey's multiple test data (*P* < 0.05).

**Figure S3.** Effects of carbohydrate synthesis on *MdATG5a*‐OE and WT apple plants after low‐light treatment. Analysis of (A) starch and (B) soluble sugar content of plants under low‐light treatment. WT, wild type, here, we used ‘GL‐3’ apple (*M. domestic*), which was also used as explants in generating transgenic apple plants; OE‐5/8/9: transgenic apple plants overexpressed *MdATG5a*, which was obtained previously. Data are means ± SD. Different letters indicated that there were significant differences in Tukey's multiple test data (*P* < 0.05).

**Figure S4.** Analysis of overall metabolomic differences in *MdATG5a*‐OE and WT apple plants after low‐light treatment. (A) Metabolite clustering heatmap analysis. (B) Types and quantities of all detected metabolites. (C) Relative contents of all metabolites. CK refers to natural light control group; LL refers to low‐light group. WT, wild type, here, we used ‘GL‐3’ apple (M. domestic), which was also used as explants in generating transgenic apple plants; OE: transgenic apple plants overexpressed *MdATG5a*, which was obtained previously.

**Figure S5.** Photosynthetic response curve of seedlings in an artificial climate light incubator.

**Figure S6.** Effects of phenotype on *MdATG5a*‐OE and WT apple plants under low light (in an artificial climate light incubator). (A) Phenotypes of WT and *MdATG5a*‐OE transgenic apple plants under low light. The scale bar = 5 cm. (B–F) Values of the leaf angle (B), plant height (C), stem diameter (D), total fresh weight (E) and total dry weight (F) in WT and *MdATG5a*‐OE transgenic apple plants under low light. WT, wild type, here, we used ‘GL‐3’ apple (*M. domestic*), which was also used as explants in generating transgenic apple plants; OE‐5/8/9: transgenic apple plants overexpressed *MdATG5a*, which was obtained previously. Data are means ± SD. Different letters indicated that there were significant differences in Tukey's multiple test data (*P* < 0.05).

**Figure S7.** EMSAs of the interaction between MdPIF7 and 5′‐biotin labeled probes.

**Figure S8.** The relative expression level of *MdPIF7* in WT under low light (A) and Dual‐luciferase reporter assay analyzing the activity of the *MdATG5a* promoter in N. benthamiana leaves after low‐light treatment (B).

**Figure S9.** Model of *MdATG5a*‐mediated autophagy improves apple performance under low light. *MdATG5a* is directly regulated by MdPIF7 in response to low‐light stress, leading to high *MdATG5a* expression and elevated autophagy activity, which are directly linked to the low‐light response of apple plants. *MdATG5a* improves photosynthetic performance mainly through four mechanisms: first, expanding leaf area to increase light‐energy capture; second, increasing the leaf N content to promote the synthesis and turnover of photosynthetic enzyme proteins and pigments; third, lowering LCP, LSP, and Rd to maximize Pn while reducing C consumption; and fourth, mitigating damage to chloroplasts and PSII to enhance light‐energy conversion efficiency in apple plants. Together, these *MdATG5a*‐mediated changes enable apple plants to accumulate more biomass and exhibit superior growth under low‐light environments.

**Table S1.** Primers used in this study.
**Table S2.** Differential metabolites in all the comparison groups.

## Data Availability

Additional data generated during this study are presented in the main text and Supporting Information of this article.
